# Uterine tumor resembling ovarian sex-cord tumor (UTROSCT) with sarcomatous features without recurrence after extended radical surgery: A case report

**DOI:** 10.1097/MD.0000000000019166

**Published:** 2020-03-13

**Authors:** Miho Sato, Mitsutake Yano, Shimpei Sato, Yoko Aoyagi, Saki Aso, Harunobu Matsumoto, Ichiro Yamamoto, Kaei Nasu

**Affiliations:** a Department of Obstetrics and Gynecology, Nakatsu Municipal Hospital; b Department of Obstetrics and Gynecology, Oita University Faculty of Medicine, Oita; c Department of Pathology, Saitama Medical University International Medical Center, Saitama; d Department of Pathology, Nakatsu Municipal Hospital; e Division of Obstetrics and Gynecology, Support System for Community Medicine, Oita University Faculty of Medicine, Oita, Japan.

**Keywords:** case report, extended radical surgery, immunohistochemistry, malignant behavior, uterine tumor resembling ovarian sex-cord tumor (UTROSCT)

## Abstract

**Rationale:**

The malignant potential and the appropriate treatment of uterine tumor resembling ovarian sex-cord tumor (UTROSCT) is controversial. Although these tumors generally have benign outcomes, several reports have described recurrences, metastases, and deaths associated with this disease.

**Patient concerns:**

A 57-year-old Japanese woman (gravida 2, para 2) was referred to our hospital for the evaluation and treatment of uterine fibroids. Magnetic resonance imaging revealed a right ovarian mass and multiple fibroids in the uterine myometrium.

**Diagnoses:**

The patient was diagnosed with UTROSCT with sarcomatous features.

**Interventions:**

She initially underwent a total abdominal hysterectomy and bilateral salpingo-oophorectomy, followed by second-stage surgery comprising pelvic and para-aortic lymphadenectomy and subtotal omentectomy.

**Outcomes:**

No postoperative recurrence was observed in the patient in 36 months.

**Lessons:**

In this case, extended radical surgery prevented the development of recurrent disease in a patient with UTROSCT with sarcomatous features. These clinicopathological findings suggest that UTROSCT is associated with several risk factors, including older age, presence of necrosis, lymphovascular invasion, significant nuclear atypia, and significant mitotic activity. This lesion type should be considered malignant and treated with curative intent.

## 1 Introduction

Uterine tumors infrequently exhibit ovarian sex cord-like features. In 1976, Clement and Scully further classified the tumors into two subgroups.^[1]^ The first subgroup, endometrial stromal tumors with sex cord-like elements (ESTSCLE), was characterized as containing focal (10%–40%) sex cord differentiation and was associated with an increased risk of recurrence and metastasis. These tumors are considered endometrial stromal tumors. Tumors in the other subgroup contain more than 50% sex cord-like cells, with no recognizable endometrial stromal component. Unlike ESTSCLEs, these uterine tumors resembling ovarian sex-cord tumor (UTROCST) are clearly separated from the surrounding tissues, exhibit distinct clinicopathological features, and are generally benign. Nevertheless, several cases of UTROSCT with recurrence and metastasis have been reported.^[2–9]^ Therefore, the malignant potential of UTROSCT, as well as the appropriate treatments, remains controversial.

The immunohistochemical expression profile of UTROSCT is polyphenotypic, and a diagnostic panel of four stains (calretinin, inhibin, CD99, and melan-A) has been proposed.^[10]^ Specifically, positivity for calretinin plus one of the other three stains is considered diagnostic.^[10]^ Researchers have attempted to identify the risk factors associated with recurrence and metastasis of UTROSCT. Moore et al reported several features associated with a UTROSCT with malignant behavior, including older age, larger mass, presence of necrosis, lymphovascular invasion, cervical involvement, significant nuclear atypia, and significant mitotic activity.^[11]^ Nevertheless, only the presence of necrosis and significant mitotic activity were statistically significant.^[11]^ Lee et al reported that older age, larger mass, and significant mitotic activity were risk factors for malignant behavior.^[12]^ Here, we report a case of UTROSCT in an older patient who exhibited the presence of necrosis, lymphovascular invasion, significant nuclear atypia, and significant mitotic activity. However, no recurrence of this tumor occurred following the extended radical surgery.

## 2 Case presentation

**Figure 1:**
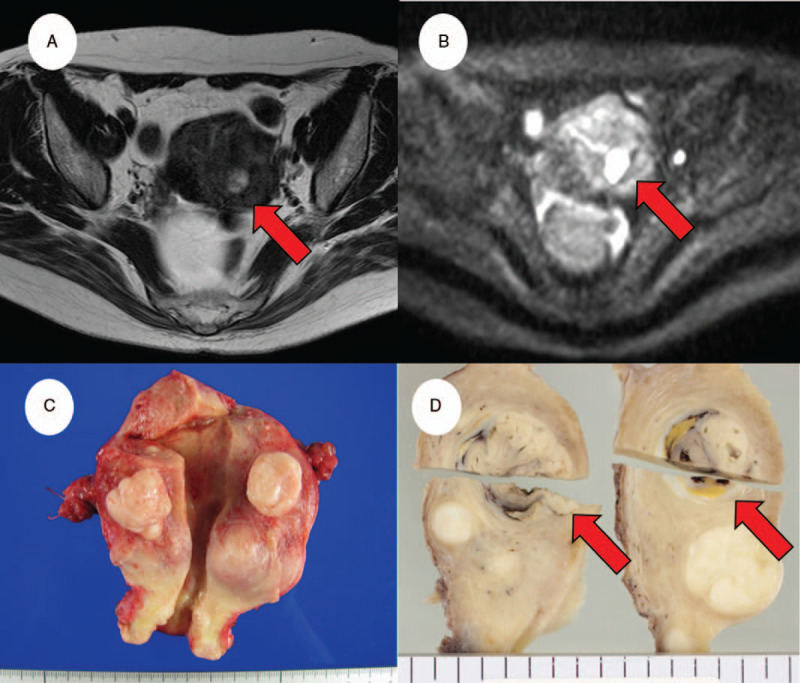
Magnetic resonance imaging (MRI) and macroscopic findings. (A) T2-weighted image and (B) diffusion-weighted image depicting high signal intensity in the uterine posterior wall fibroid (red arrow). (C) No mass is visible in the cavity of the resected uterus. (D) Sagittal cut sections. A myometrial mass (red arrows) in the posterior of the uterine body measured 25 mm × 20 mm and had a mixed-color (white, yellow, and black) cut surface.

A 57-year-old Japanese woman (gravida 2, para 2) had previously visited another clinic because of lower abdominal pain. A right ovarian mass and multiple uterine masses were detected using transvaginal ultrasonography, and she was referred to our hospital for evaluation and treatment. She had a history of hypertension and a family history of stomach cancer (father) and uterine leiomyomas and auditory tumor (mother). Magnetic resonance imaging (MRI) revealed a 50-mm mass in the right ovary and multiple fibroids in the uterine myometrium that were suspected to be an ovarian serous adenoma and uterine leiomyomas, respectively (Fig. 1A,B). No metastasis was detected on a systemic computed tomography scan. The serum levels of the tumor markers cancer antigen 125, carbohydrate antigen 19-9, and cancer antigen 72-4 were 11.5, 11.5, and 9.4 U/mL, respectively. Cervical and endometrial cytology were negative for neoplastic lesions.

**Figure 2:**
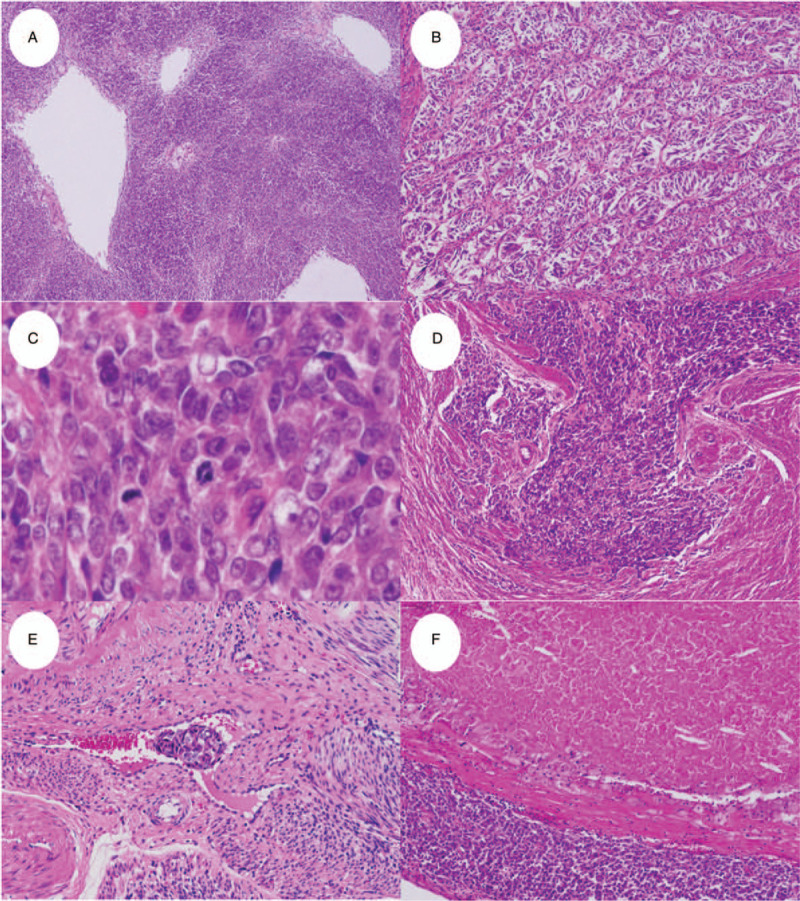
Histology of the uterine tumor resembling ovarian sex-cord tumor (UTROSCT). The tumor exhibited a variety of histologic patterns including (A) sheets and follicle-like, (B) cord-like, and tubular structures. (C) The tumor contained significant nuclear atypia and mitotic activity. (D) Myometrial invasion, (E) lymphovascular invasion, and (F) necrosis were detected. Magnification: 20× for all but (C), 100×.

The patient underwent a total abdominal hysterectomy and bilateral salpingo-oophorectomy. The uterine body contained a tumor measuring 25 mm × 20 mm located in the posterior wall that was not continuous with the endometrium. It had a mixed white, yellow, and black cut surface (Fig. 1C, D). Histologically, the tumor exhibited proliferating spindle- to oval-shaped cells with non-uniform nuclei, distinct nucleoli, and eosinophilic or scant cytoplasm arranged in sheets, follicles, fascicles, cords, and a tubular pattern (Fig. 2A, B). The majority of the tumor cells appeared mesenchymal in origin but did not appear to be endometrial stroma. Frequent mitotic figures were observed in the sarcomatous fascicular components (15–20/10 high-power fields; Fig. 2C). Myometrial invasion, lymphovascular invasion, necrosis, and hemorrhage were detected (Fig. 2D–F). No associated endometrial lesions were seen. The other uterine fibroids and the right ovarian tumor were diagnosed as leiomyomas and serous adenoma, respectively. No other neoplasias were present in the uterus or adnexa.

**Figure 3:**
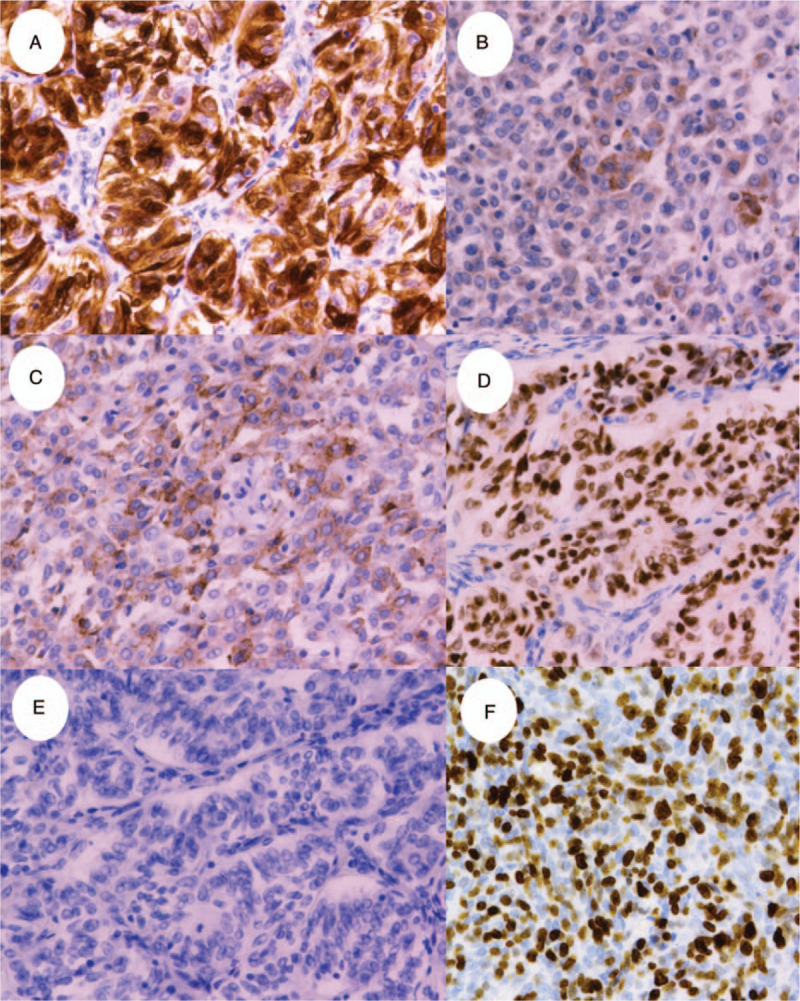
Immunohistochemistry of the uterine tumor resembling ovarian sex-cord tumor (UTROSCT). The tumor was positive for (A) calretinin (diffuse), (B) α-inhibin (focal), (C) CD99, and (D) progesterone receptor, but negative for melan-A (E). (F) The MIB-1 labeling index was 70%. Magnification: 40×.

An immunohistochemical analysis of the surgical sample revealed that the tumor cells were positive for calretinin (diffuse), α-inhibin (focal), CD99 (focal), AE1/AE3 (diffuse), CD10 (focal), estrogen receptor (diffuse), progesterone receptor (diffuse), S-100 protein (diffuse), vimentin (diffuse), CD56 (focal), chromogranin A (focal), and synaptophysin (very focal; Fig. 3A–D). The cells were negative for FOXL2, melan-A, epithelial membrane antigen, α-smooth muscle actin, desmin, human melanoma black-45, CD34, c-kit, and h-caldesmon (Fig. 3E). The MIB-1 labeling index was approximately 70% in the hot spot (Fig. 3F). Based on these findings, the patient was diagnosed with a UTROSCT with sarcomatous features. She underwent two-stage surgery (pelvic and para-aortic lymphadenectomy and subtotal omentectomy). Pathologically, the tumor had not extended into the cervix, adnexa, resected lymph nodes, or omentum. No recurrence was observed in the patient since the first surgery (39 months). We obtained written informed consent from the patient for publication.

## 3 Discussion

UTROSCT is a controversial disease entity, as several reports have described recurrences, metastases, and deaths associated with these usually benign tumors. Moore et al reported that 8 of 34 patients (23.5%) with UTROSCT developed extrauterine metastases to a variety of sites, including the pelvic and abdominal peritoneum, ovary, lymph nodes, bone, liver, and lung. Moreover, three patients (8.8%) died as a result of the tumor.^[11]^ We reviewed these reported cases of UTROSCTs with malignant behavior (Table 1),^[2–9]^ and found that patients who underwent extended radical surgery did not develop recurrences. In contrast, hysterectomy alone was associated with a higher rate of recurrence. Therefore, extended radical surgery, including bilateral salpingo-oophorectomy, lymphadenectomy, and omentectomy, may reduce the rate of recurrence among cases of aggressive UTROSCTs.

**Table 1:**
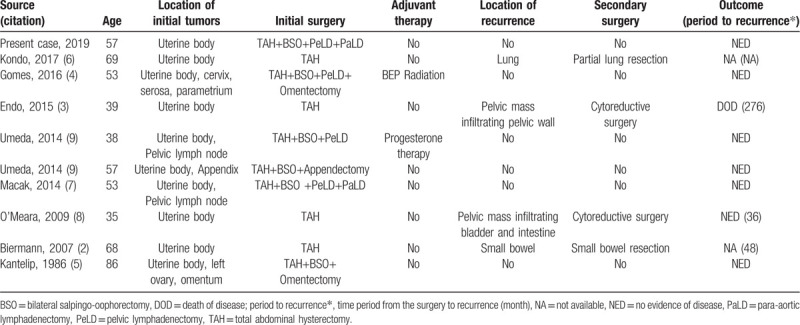
Summary of aggressive UTROSCT cases.

In previous studies of UTROSCTs, malignant behavior was associated with older age, larger tumors, the presence of necrosis, lymphovascular invasion, cervical involvement, significant nuclear atypia, and significant mitotic activity.^[11]^ In particular, the presence of necrosis and significant mitotic activity was statistically significant.^[11]^ The present case exhibited mitotic activity, a high MIB-1 labeling index, necrosis, and lymphovascular invasion and hence, was considered high-risk for recurrence. Nevertheless, the patient underwent a second extended radical surgery, including lymphadenectomy, and progressed without recurrence. These results suggest that, for a high-risk UTROSCT, both hysterectomy and extended radical surgery, including salpingo-oophorectomy, parametrium resection, and pelvic and para-aortic lymphadenectomy, should be considered. Although no effective chemotherapy regimen has been identified, high-dose progesterone therapy may be effective for UTROSCTs which often exhibit diffuse positivity for hormone receptor expression.^[9]^

UTROSCT is challenging because it is often detected incidentally after surgery for other benign lesions.^[13]^ For this reason, the initial treatment often does not involve sufficiently extensive surgery. Because UTROSCTs are often located within the uterine myometrium and do not involve the endometrium, they are not detected by endometrial biopsy. Takeuchi et al reported that UTROSCTs exhibited high signal intensity on MRI diffusion-weighted images in contrast to the generally low signal associated with leiomyoma. Therefore, this modality is useful for a differential diagnosis.^[14]^ A combination of diffusion-weighted imaging and apparent diffusion coefficient maps are useful for distinguishing benign leiomyomas from leiomyosarcomas and smooth muscle tumors of uncertain malignant potential.^[15]^ Because the tumor in the present case exhibited high signal intensity on MRI diffusion-weighted images, we should have considered the possibility of malignancy. The initial surgery led to a diagnosis of UTROSCT, and a second-stage extended surgery was performed. We believe that second-stage surgery should not be avoided when a UTROSCT associated with several risk factors is encountered.

This study was limited by a lack of molecular analysis. UTROSCTs lack the *JAZF1-SUZ12* fusion that is characteristic of endometrial stromal tumors, indicating that these lesions are unlikely to be of endometrial stromal origin.^[16]^ Instead, we confirmed the presence of the characteristic immunohistochemical features of UTROSCT, the absence of morphological features of the endometrial stroma, and the absence of continuity with the endometrium.

In conclusion, we encountered a patient with UTROSCT with sarcomatous features who was successfully treated by extended surgery and did not develop recurrent disease. These clinicopathological findings suggest that a UTROSCT associated with several risk factors (e.g., older age, the presence of necrosis, lymphovascular invasion, significant nuclear atypia, and significant mitotic activity) should be considered a malignant tumor and treated with curative intent.

## Acknowledgments

We thank Tomomi Katoh and Masanori Yasuda, Department of Pathology, Saitama Medical University International Medical Center, for their technical support. We also thank Editage (www.editage.jp) for English language editing.

## Author contributions

MS: conception and writing of the manuscript. SS, YA, SA, HM: collection of clinical data. IY: pathologic diagnosis and immunohistochemical analysis. KN: collection of clinical data and revision of the manuscript. MY: conception, pathologic diagnosis, immunohistochemical analysis, and revision of the manuscript. All authors read and approved the final manuscript prior to submission.

Mitsutake Yano orcid: 0000-0002-4436-838X.

## References

[1] Clement PB Scully RE Uterine tumors resembling ovarian sex-cord tumors. A clinicopathologic analysis of fourteen cases. Am J Clin Pathol 1976;66:512–25. 961630 10.1093/ajcp/66.3.512

[2] Biermann K Heukamp LC Buttner R Uterine tumor resembling an ovarian sex cord tumor associated with metastasis. Int J Gynecol Pathol 2008;27:58–60. 18156976 10.1097/pgp.0b013e318057faf5

[3] Endo D Todo Y Okamoto K A case of recurrent group II uterine tumor resembling ovarian sex-cord tumors, against which two hormonal agents were ineffective. Taiwan J Obstet Gynecol 2016;55:751–3. 27751432 10.1016/j.tjog.2015.04.008

[4] Gomes JR Carvalho FM Abrao M Uterine tumors resembling ovarian sex-cord tumor: A case-report and a review of literature. Gynecol Oncol Rep 2016;15:22–4. 26937483 10.1016/j.gore.2015.11.003 PMC4750014

[5] Kantelip B Cloup N Dechelotte P Uterine tumor resembling ovarian sex cord tumors: report of a case with ultrastructural study. Human Pathol 1986;17:91–4. 3943854 10.1016/s0046-8177(86)80161-7

[6] Kondo Y Sakaguchi S Mikubo M Lung metastases of a uterine tumor resembling ovarian sex-cord tumor: Report of a rare case. Diagn Cytopathol 2018;46:88–91. 28869333 10.1002/dc.23804

[7] Macak J Dundr P Dvorackova J Uterine tumors resembling ovarian sex cord tumors (UTROSCT). Report of a case with lymph node metastasis. Cesk Patol 2014;50:46–9. 24624987

[8] O’Meara AC Giger OT Kurrer M Case report: recurrence of a uterine tumor resembling ovarian sex-cord tumor. Gynecol Oncol 2009;114:140–2. 19406461 10.1016/j.ygyno.2009.03.021

[9] Umeda S Tateno M Miyagi E Uterine tumors resembling ovarian sex cord tumors (UTROSCT) with metastasis: clinicopathological study of two cases. Int J Clin Exp Pathol 2014;7:1051–9. 24696722 PMC3971308

[10] Irving JA Carinelli S Prat J Uterine tumors resembling ovarian sex cord tumors are polyphenotypic neoplasms with true sex cord differentiation. Mod Pathol 2006;19:17–24. 16118629 10.1038/modpathol.3800475

[11] Moore M McCluggage WG Uterine tumour resembling ovarian sex cord tumour: first report of a large series with follow-up. Histopathology 2017;71:751–9. 28656712 10.1111/his.13296

[12] Lee CH Kao YC Lee WR Clinicopathologic characterization of GREB1-rearranged uterine sarcomas with variable sex-cord differentiation. Am J Surg Pathol 2019;43:928–42. 31094921 10.1097/PAS.0000000000001265

[13] Blake EA Sheridan TB Wang KL Clinical characteristics and outcomes of uterine tumors resembling ovarian sex-cord tumors (UTROSCT): a systematic review of literature. Eur J Obstet Gynecol Reprod Biol 2014;181:163–70. 25150955 10.1016/j.ejogrb.2014.07.050

[14] Takeuchi M Matsuzaki K Bando Y A case of uterine tumor resembling ovarian sex-cord tumor (UTROSCT) exhibiting similar imaging characteristics to those of ovarian sex-cord tumor. Magn Reson Med Sci 2019;18:113–4. 29794407 10.2463/mrms.ci.2017-0177 PMC6460130

[15] Lin G Yang LY Huang YT Comparison of the diagnostic accuracy of contrast-enhanced MRI and diffusion-weighted MRI in the differentiation between uterine leiomyosarcoma /smooth muscle tumor with uncertain malignant potential and benign leiomyoma. J Magn Reson Imaging 2016;43:333–42. 26383110 10.1002/jmri.24998

[16] Staats PN Garcia JJ Dias-Santagata DC Uterine tumors resembling ovarian sex cord tumors (UTROSCT) lack the JAZF1-JJAZ1 translocation frequently seen in endometrial stromal tumors. Am J Surg Pathol 2009;33:1206–12. 19542872 10.1097/PAS.0b013e3181a7b9cf

